# Association of Online Learning Behavior and Learning Outcomes for Medical Students: Large-Scale Usage Data Analysis

**DOI:** 10.2196/13529

**Published:** 2019-08-21

**Authors:** Martina Bientzle, Emrah Hircin, Joachim Kimmerle, Christian Knipfer, Ralf Smeets, Robert Gaudin, Peter Holtz

**Affiliations:** 1 Leibniz-Institut für Wissensmedien (Knowledge Media Research Center) Tübingen Germany; 2 AMBOSS GmbH Cologne Germany; 3 Department of Psychology Eberhard Karls University Tübingen Germany; 4 Department of Oral Maxillofacial Surgery University Medical Center Hamburg-Eppendorf Hamburg Germany; 5 Laboratory for Innovation Science at Harvard Harvard University Cambridge, MA United States; 6 Department of Oral- and Maxillofacial Surgery Charité-Universitätsmedizin Berlin Berlin Germany

**Keywords:** learning engagement, medical online learning platform, big data analytics, writing notes, learning outcomes

## Abstract

**Background:**

Digital learning environments have become very common in the training of medical professionals, and students often use such platforms for exam preparation. Multiple choice questions (MCQs) are a common format in medical exams and are used by students to prepare for said exams.

**Objective:**

We aimed to examine whether particular learning activities contributed more strongly than others to users’ exam performance.

**Methods:**

We analyzed data from users of an online platform that provides learning materials for medical students in preparation for their final exams. We analyzed whether the number of learning cards viewed and the number of MCQs taken were positively related to learning outcomes. We also examined whether viewing learning cards or answering MCQs was more effective. Finally, we tested whether taking individual notes predicted learning outcomes, and whether taking notes had an effect after controlling for the effects of learning cards and MCQs. Our analyses from the online platform Amboss are based on user activity data, which supplied the number of learning cards studied and test questions answered. We also included the number of notes from each of those 23,633 users who had studied at least 200 learning cards and had answered at least 1000 test exam questions in the 180 days before their state exam. The activity data for this analysis was collected retrospectively, using Amboss archival usage data from April 2014 to April 2017. Learning outcomes were measured using the final state exam scores that were calculated by using the answers voluntarily entered by the participants.

**Results:**

We found correlations between the number of cards studied (*r*=.22; *P*<.001) and the number of test questions that had been answered (*r*=.23; *P*<.001) with the percentage of correct answers in the learners’ medical exams. The number of test questions answered still yielded a significant effect, even after controlling for the number of learning cards studied using a hierarchical regression analysis (*β*=.14; *P*<.001; *ΔR*^2^=.017; *P*<.001). We found a negative interaction between the number of learning cards and MCQs, indicating that users with high scores for learning cards and MCQs had the highest exam scores. Those 8040 participants who had taken at least one note had a higher percentage of correct answers (80.94%; SD=7.44) than those who had not taken any notes (78.73%; SD=7.80; *t*_23631_=20.95; *P*<.001). In a stepwise regression, the number of notes the participants had taken predicted the percentage of correct answers over and above the effect of the number of learning cards studied and of the number of test questions entered in step one (*β*=.06; *P*<.001; *ΔR*^2^=.004; *P*<.001).

**Conclusions:**

These results show that online learning platforms are particularly helpful whenever learners engage in active elaboration in learning material, such as by answering MCQs or taking notes.

## Introduction

### Background

**Figure 1:**
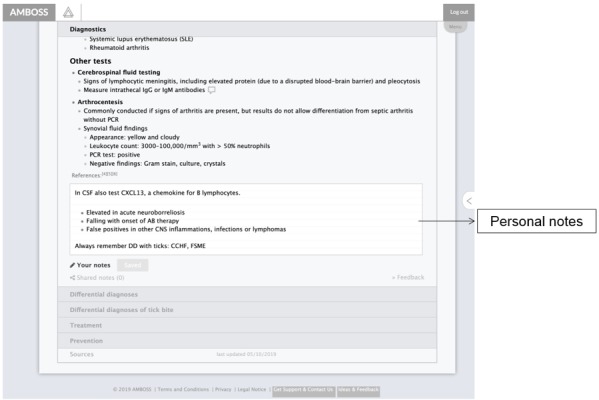
Example of personal notes on a learning card from the AMBOSS platform.

Digital learning environments are used with increasing frequency in medical education [1]. They are often integrated as teaching formats into the didactic concept of medical studies [2-4] and are also extensively used by students for exam preparation [5,6]. Multiple choice questions (MCQs) are a common format for medical exams and are therefore preferentially used by students to prepare for their medical exams [7]. In response to the high demand for practicing with MCQs, several online platforms that provide students with both medical information and the opportunity to answer MCQs relevant to various exams have become available. These platforms also give immediate feedback on the correctness of their answers. A prominent example of such a platform is AMBOSS [8], which is provided by the company AMBOSS, and is available in both English and German. The central concept of the platform is to provide MCQs that are linked to extensive medical information needed to answer relevant exam questions correctly (learning cards). Thus, the platform connects textbook content directly to the common format of MCQs used in the actual final exam. In addition, the platform offers the option of taking personal notes about the learning content. These personal notes can be written directly onto the corresponding learning cards on the computer screen (Figure 1).

The first and second parts of medical studies in Germany are completed by taking two final state exams (1st and 2nd Staatsexamen) that are made up of MCQs. The AMBOSS platform provides its users with the MCQs used in the final state exams in recent years. On the official exam days, AMBOSS provides a preliminary statistical prognosis of an individual’s real exam results in cooperation with the learning platform Medi-learn [9]. In order to use this service, students enter their answers from their actual final exams into the platform to get immediate feedback on the number of correct answers. In the study presented here, we used the results of the participants’ second state medical exam, voluntarily provided by them, to measure learning performance. One aim of the study was to apply insights from educational psychology to the setting of an online learning platform in order to test specific hypotheses with a large sample of medical students. We also aimed to examine whether particular learning activities contributed more strongly than others to users’ exam performance. User activity records and their comparison to actual final exam results were utilized to achieve these research goals.

### Impact of Engagement on Learning

There is a long tradition of research dealing with the influence of learning activities on learning outcomes [10-12]. Time spent on learning is a predictor of learning outcomes in both offline and online settings [13]. The time spent with actual learning activities as opposed to time merely being present in a certain setting is particularly important [10,12]. Thus, hypothesis 1 was that the number of learning cards viewed are positively related to learning outcomes.

It is also known that active cognitive engagement with learning material is an essential factor of learning. Active learning as opposed to only passively receiving information increases students’ performance [14,15]. One way to engage in learning actively is to answer MCQs. Therefore, hypothesis 2 was that the numbers of MCQs answered are positively related to learning outcomes.

As an exploratory research question, we also examined whether viewing learning cards or answering MCQs was more effective in terms of learning outcomes.

Another way to elaborate on learning material is to take individual notes on the learning content [16]. Taking notes can have several advantages [17,18], such as that, in many cases, note taking demands that learners make a connection with their previous knowledge (encoding benefit). In addition, learners also have the opportunity to study their notes after they have made them (external storage). Based on these considerations, hypothesis 3a was that better learning outcomes are shown for learners who took notes than for learners who did not take notes.

Moreover, we assumed that the level of engagement in taking notes had an influence on learning over and above the effect of other general learning activities. Thus, hypothesis 3b was that the numbers of notes the learners had taken would predict the learning outcome even when controlling for the effects of answering MCQs and studying learning cards.

## Methods

**Figure 2:**
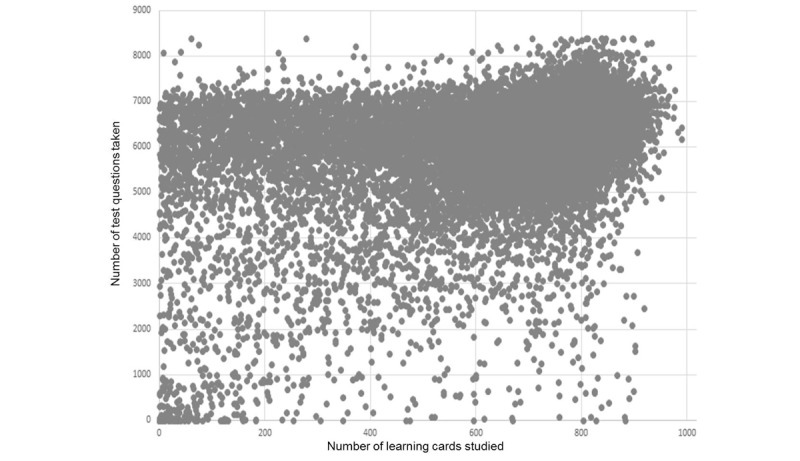
Distribution of the numbers of learning cards studied and test questions taken among the participants.

For the present study, the data of AMBOSS users who had taken their final state medical exams between October 2014 and April 2017 was evaluated. Users were included in the analysis if they had entered the results of their exams, had previously opened at least 200 learning cards, and had answered at least 1000 MCQs, resulting in a sample of 23,633 AMBOSS users (for the CONSORT flow diagram see Multimedia Appendix 1). This procedure eliminated users who did not seriously use AMBOSS for exam preparation (Figure 2), while keeping as many usable cases as possible to represent a wide range of different usage patterns. Learning cards, test questions, and notes for all accepted users were utilized in further analyses (Textbox 1).

**Learning features included in the analysis.**

Learning cards:

- The number of unique learning cards that were opened by the user.

Test questions (MCQs: multiple choice questions):

- The number of unique test questions that were answered by the user.

Notes

- The number of a user’s notes that refer to a specific learning card that comprised five or more characters (a threshold of five characters was chosen to exclude notes that only served the function of marking a learning card as read).

**Figure 3:**
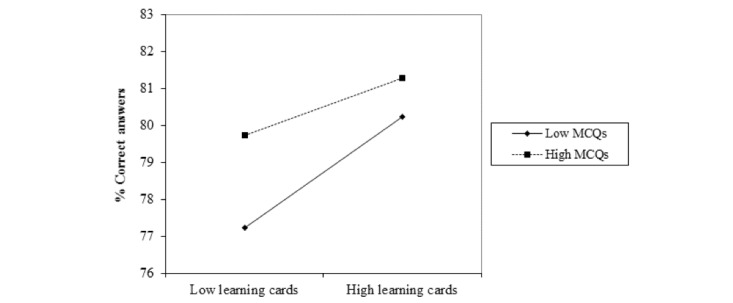
Interaction between the number of learning cards and the number of test questions (MCQs). The dependent variable is the percentage of correct answers in the final exam. MCQs: multiple choice questions.

We used the percentage of correct answers in the final state exam, as entered by the participants, as our main dependent variable. All statistical analyses were done with SPSS 22. The interaction analysis (see Table 1 and Figure 3) was done with the Microsoft Excel Macro from Jeremy Dawson’s website [19,20].

Data retrieval for this study was conducted with permission of registered users of AMBOSS who agreed to the usage and privacy terms in the registration process. The AMBOSS system generates usage data about accessing MCQs, using learning cards, and taking notes to provide statistical analysis functions to its users. The data gathered is analyzed to give individual users recommendations for their learning. Besides individual recommendations, anonymous usage data is analyzed in user research settings to improve the quality of the product. AMBOSS agreed to share its anonymous archival data while preserving the privacy of individual user data, according to the rules of General Data Protection Regulation in Germany (Datenschutzgrundverordnung [DSGVO]). This procedure is in line with the requirements of the local ethics committee.

**Table 1:** Hierarchical regression analysis with the number of learning cards and test questions (MCQs; Step 1) and the interaction between the two (Step 2) as independent variables, and the percentage of correct answers as dependent variable.

		*B*	*SE B*	*β*	*ΔR* ^2^
**Step 1**					.067^a^
	Learning cards	0.747	0.038	.14^a^	—^b^
	Test questions	1.094	0.054	.20^a^	—
**Step 2**					.001^a^
^ ^	Learning cards × Test questions^c^	0.064	0.016	.05^a^	—

^a^This denotes a value with *P*<.001.

^b^Not applicable.

^c^Learning cards and test questions (MCQs) were z-standardized for the analysis.

## Results

### Descriptives

On average, the users had studied 645.91 learning cards (SD=222.06) and answered 5981.87 test questions (SD=1309.52). A total of 8040 users took at least one note, with a mean of 94.31 (SD=293.89). In addition, users reported an average of 79.48% (SD=7.75) correct answers in their state exams.

### The Number of Learning Cards and Multiple Choice Questions as Predictors of Learning Outcomes

Both the number of learning cards studied (*r*=.22; *P*<.001) and the number of MCQs answered *(r=.* 23, *P*<.001) were substantially correlated with the percentage of correct answers in the state exam. We used hierarchical regression analysis to answer the question as to whether the number of MCQs answered explained the percentage of correct answers in the exam over and above the number of learning cards studied. The number of test questions answered still yielded a significant effect even after controlling for the number of learning cards studied (see Table 2, step 2).

**Table 2:** Hierarchical regression analysis with the number of learning cards (Step 1), test questions (Step 2), and notes (Step 3) as independent variables and the percentage of correct answers as dependent variable.

		*B*	*SE B*	*β*	*ΔR* ^2^
**Step 1**					.043^a^
	Learning cards	0.011	0	.21^a^	—^b^
**Step 2**					.017^a^
	Learning cards	0.008	0	.16^a^	—
	Test questions	0.001	0	.14^a^	—
**Step 3**					.004^a^
	Learning cards	0.008	0	.15^a^	—
	Test questions	0.001	0	.14^a^	—
	Notes	0.003	0	.06^a^	—

^a^This denotes a value with *P*<.001.

^b^Not applicable.

### The Interaction Between Multiple Choice Questions and Learning Cards

As a means of answering the exploratory research question on the relative effectiveness of learning cards and MCQs, we calculated a moderated regression analysis [21] in order to analyze possible interaction effects. We found a small, albeit significant, negative interaction between the numbers of learning cards and MCQs, indicating that those users who neither studied learning cards nor took MCQs scored worse in their final exam. Users with high scores for learning cards as well as for MCQs had the highest scores (see Table 1 and Figure 3).

### The Number of Notes as a Predictor of Learning Outcomes

A two-tailed independent sample t-test showed that those 8040 participants who had taken at least one note had a higher percentage of correct answers (80.94%; SD=7.44) than those who had not taken notes (78.73%; SD=7.80; *t*_23631_=20.95; *P*<.001).

A stepwise regression showed that the number of notes the participants had taken still predicted the percentage of correct answers over and above the number of learning cards studied and the numbers of MCQs answered (see Table 2, Step 3).

## Discussion

### Principal Findings

Engaging with online learning materials, in the form of studying learning cards or answering test questions, was related to positive learning outcomes reflected in final grades on a state medical exam. Combining the learning activities of reading learning cards and answering MCQs resulted in the highest test scores on the final exams. The integration of both features on one learning platform appears to be a good way to support the learning activities of medical students. Moreover, taking electronic notes also went along with a higher percentage of correct answers on the medical exams. This finding held even when controlling for the effect of a number of other learning activities. Presumably, taking notes led to a deeper understanding of the learning material and hence to better retention.

### Limitations

AMBOSS was originally created specifically for medical students in Germany, but there is also an English version available. It is possible that the combination of using learning cards and answering MCQs could be helpful for exam preparation in general. As MCQs are a common examination format not only in Germany but also in other countries, it is evident that our study questions are also relevant to other places in the world. Therefore, the question of how widely our findings can be generalized to other online platforms in different educational systems remains open. More research is needed to assess the robustness and generalizability of our main findings.

It is an established finding that time spent on learning can be a predictor of learning outcomes. For technical reasons, it was not possible to control for time spent in our analysis. Future studies should take this variable into account to differentiate between the impact of time spent on learning and the impact of specific learning activities, such as answering MCQs, reading learning cards, and writing notes. Another question for future research will be to figure out how tools that allow learners to share their knowledge, and mutually support each other [22-24], can improve the effectiveness of online learning platforms in the field of medicine.

However, we cannot rule out the possibility that the relationship between a greater use of learning cards, test questions, and notes with better performance on a test may be influenced by another variable. For example, it may be that medical students with a particularly high level of achievement motivation like to use these learning opportunities and are at the same time the ones who already perform better. Future studies could address this limitation by, for example, allowing one group of students to make use of those tools and comparing them with those who did not have this opportunity. Finally, the question arises of whether or not the type of examination performance recorded here is in fact a good indicator of knowledge acquisition. MCQs are highly controversial in this respect. Students who have learned a lot with MCQs are better in the exam, which also uses MCQs, but whether this better performance leads to better, actually applicable knowledge has not yet been fully clarified.

### Conclusions

Online resources can play an important role in the training of medical professionals [25-27]. Studying learning material online is more effective whenever learning platforms offer their users ways of individualizing their environment [28] and of engaging more deeply with the learning topics [29]. One way of engaging more deeply could involve, for example, providing practice test questions or the technical means of attaching individual notes to the learning materials, which would reinforce the learning process.

## Appendix

Multimedia Appendix 1 Consort flow diagram.

## Abbreviations

DSGVO: Datenschutzgrundverordnung
GmbH: Gesellschaft mit beschränkter Haftung
MCQ: multiple choice question
