# Supplementary figures and images for: Association of Online Learning Behavior and Learning Outcomes for Medical Students: Large-Scale Usage Data Analysis
Source: JMIR Med Educ. 2019 Aug 21;5(2):e13529. doi: 10.2196/13529 (PMC6724501; doi:10.2196/13529)

## CONSORT FLOW DIAGRAM

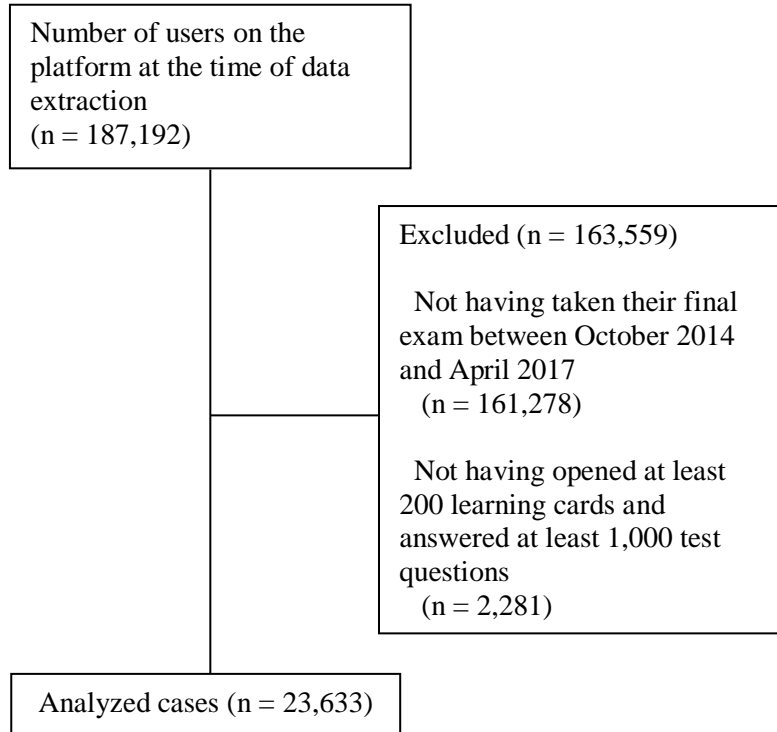

Supplement: Multimedia Appendix 1 [file mededu_v5i2e13529_app1.pdf]
